# Decoding Market Voices: Beyond the Charts Analysis of Cryptocurrency Market Microstructure

**DOI:** 10.12688/f1000research.173888.1

**Published:** 2026-02-16

**Authors:** Nitika Sharma, Sridhar Manohar, A B Satish Rao

**Affiliations:** 1 Chitkara University Chitkara Business School, Chandigarh, Chandigarh, India; 2 Chitkara University Chitkara Business School, Chandigarh, Chandigarh, India; 3 Department of Hotel Administration and Hospitality, Welcomgroup Graduate School of Hotel Administration, Manipal Academy of Higher Education, Manipal, India

**Keywords:** Podcast analysis, Descending Hierarchical Classification, Factorial Analysis, Lexicometric, Iramuteq, Market Microstructure, Cryptocurrencies

## Abstract

**Background:**

As the financial markets are becoming increasingly complicated, there is a need to understand the evolving market microstructure landscape. While the existing research is well-focused on quantitative aspects of financial markets, qualitative aspects remain underexplored. The purpose of this study is to explore the market microstructure of cryptocurrencies through a mixed-methodological approach.

**Methods:**

By leveraging podcast analysis as primary data source, the study aims to uncover core thematic dimensions through techniques of lexicometry using Iramuteq. A total of 12 domain-specific podcasts are analyzed and processed in the study for descending hierarchical classification, resulting in five lexical clusters. These clusters are then subjected to factorial analysis using the Scikit-learn library in Python (v3.10).

**Results:**

The descending hierarchical classification reveals five thematic clusters reflecting institutional infrastructure, market foundation, efficiency and resilience, forecasting and modelling dynamics, and market structure and efficiency. Followed by this, the factorial analysis discloses three dominant factors; market structure, forecasting models, and resilience mechanisms that shape the understanding of cryptocurrency market microstructure. The findings indicate that discussions on market microstructure of cryptocurrencies are multidimensional and are closely linked with both technological and regulatory developments.

**Conclusion:**

The study contributes to literature by introducing a novel data source for qualitative finance research. The use of podcasts enhances the depth of extracted themes. Practitioners and academicians can gain updated insights on evolving market microstructure mechanisms for digital financial markets. The originality of this study lies in its unique approach, bridging top industry expert insights with quantitative techniques to offer a deeper understanding and future direction in market microstructure of cryptocurrencies.

## 1. Introduction

Global financial markets have experienced numerous revolutions, showing rapid growth over the years introducing new avenues for investment ( Gomber et al. 2018). The traditional banking infrastructure has been altered by the 24/7 trading landscape of cryptocurrencies ( Baur et al. 2018). The cryptocurrency market has grown rapidly reaching peak capitalization of over $3.70 trillion in 2024 with numerous digital assets being traded across decentralized exchanges. These rapid expansions have spurred innovation in technology with the growth in investment avenues, allowing investors to invest with ease and sophistication. It has attracted a diverse set of market participants, including institutional investors, high-frequency traders, and retail investors. However, the increasing adoption has introduced complexities in the cryptocurrency market along with challenges in understanding its microstructure ( Almeida and Gonçalves 2024).

**Figure 1.**
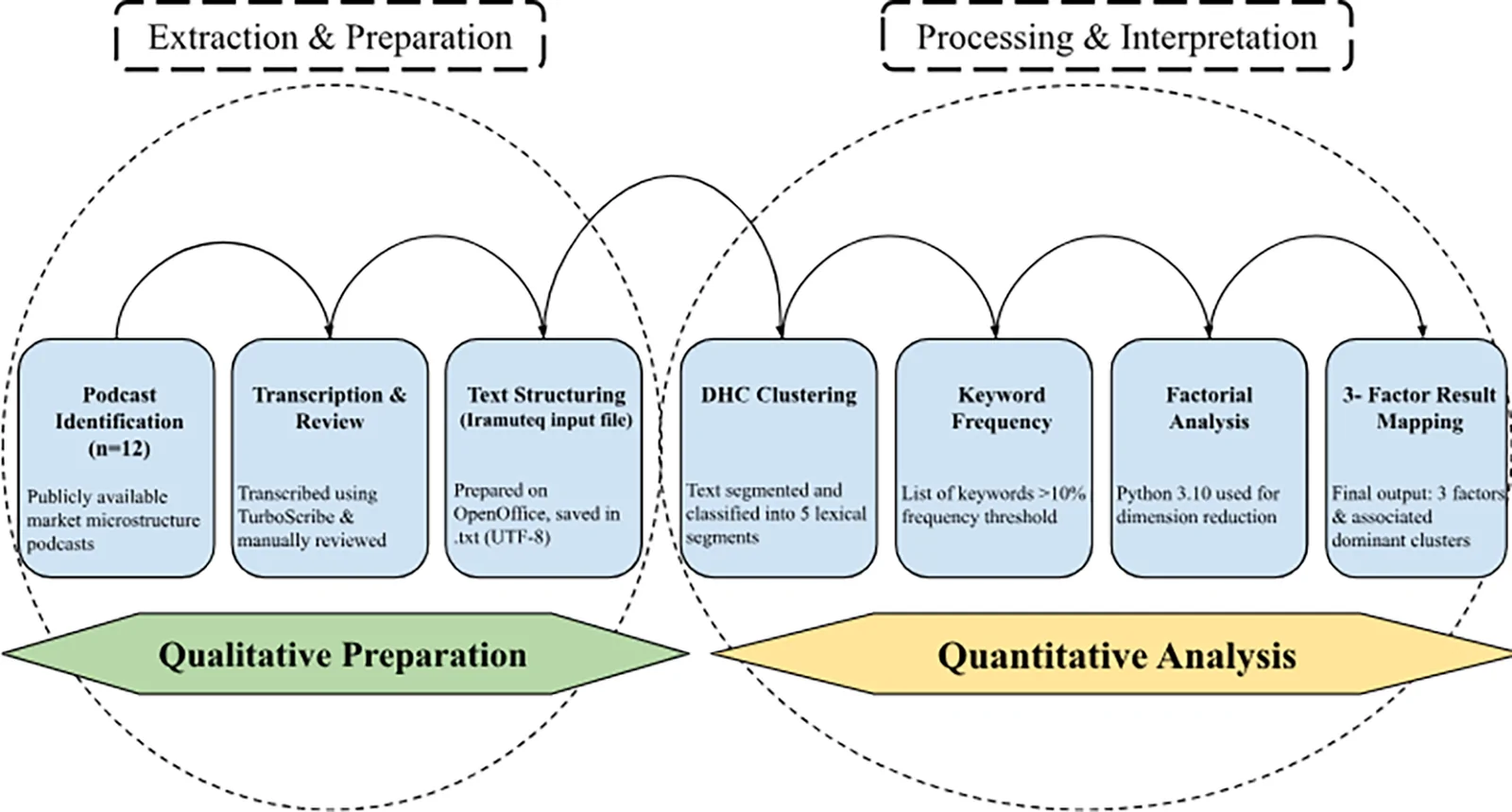
Data processing and analysis. [Source: Author’s own work].

Unlike the traditional markets landscape, cryptocurrency markets comprise unique microstructure characteristics ( Hairudin et al. 2022). It includes a number of challenges, including fragmented liquidity, information asymmetry, extreme volatility shocks, and much more ( Makarov and Schoar 2020). Key aspects of market analysis such as bid-ask-spread, order book, tick size, and transaction cost vary widely compared to traditional setting, often diverging from patterns and trends ( Feng et al. 2022). These elements altogether define market microstructure, the study of financial markets and how they operate ( Easley and O’Hara 1995). Understanding microstructure mechanisms is crucial for assessing systematic risks, eradicating market manipulation, and for sophisticated order execution ( Abrol et al. 2016).

The microstructure of cryptocurrencies present varied challenges that differ them from other asset classes. For instance, the FTX collapse of 2022 revealed critical vulnerabilities in market structure, including mismatches in liquidity, vague order matching, and the absence of circuit breakers ( Conlon et al. 2023). Consequently, such collisions in the market require an in-depth analysis of market microstructure of cryptocurrencies. Despite evolving research in cryptocurrencies, literature remains limited on exploring its microstructure, majorly relying on theoretical frameworks designed for traditional markets. The disadvantage of reliance on these frameworks is their limitation in factors that account for crypto-specific phenomena.

Existing literature has laid scattered opinions on various aspects of the cryptocurrency market. Some authors indicate the decrease in liquidity results in increased efficiency ( Le Tran and Leirvik 2020; López-Martín et al. 2021; Brauneis et al. 2021); while others indicate that liquidity has no role to play in the efficiency of market since it is caused as per overall market situations ( Caporale et al. 2018; Keshari Jena and Dash 2020). Research has focused on technological aspects for price prediction through availability of detailed electronic records ( Sirignano & Cont 2021). On the other hand, studies have focused on information symmetry using market microstructure to get a true picture of market dynamics ( Said et al. 2024). Bhat et al. (2024) proposed a hybrid model consisting of macroeconomic fundamentals and market microstructure variables for price predictions over a short to medium investment period. Despite rich insights on certain aspects of market mechanism, significant gaps persist in explaining how the decentralized system reshape microstructure dynamics. For instance, while the traditional models assume centralized price formation, cryptocurrency markets demonstrate price discovery across fragmented venues with different levels of transparency ( Albers et al. 2021). These changes in technology impose new challenges and opportunities, particularly in comprehending the mechanisms of market and process for establishment of prices leading to microstructural complexities.

**Figure 5.**
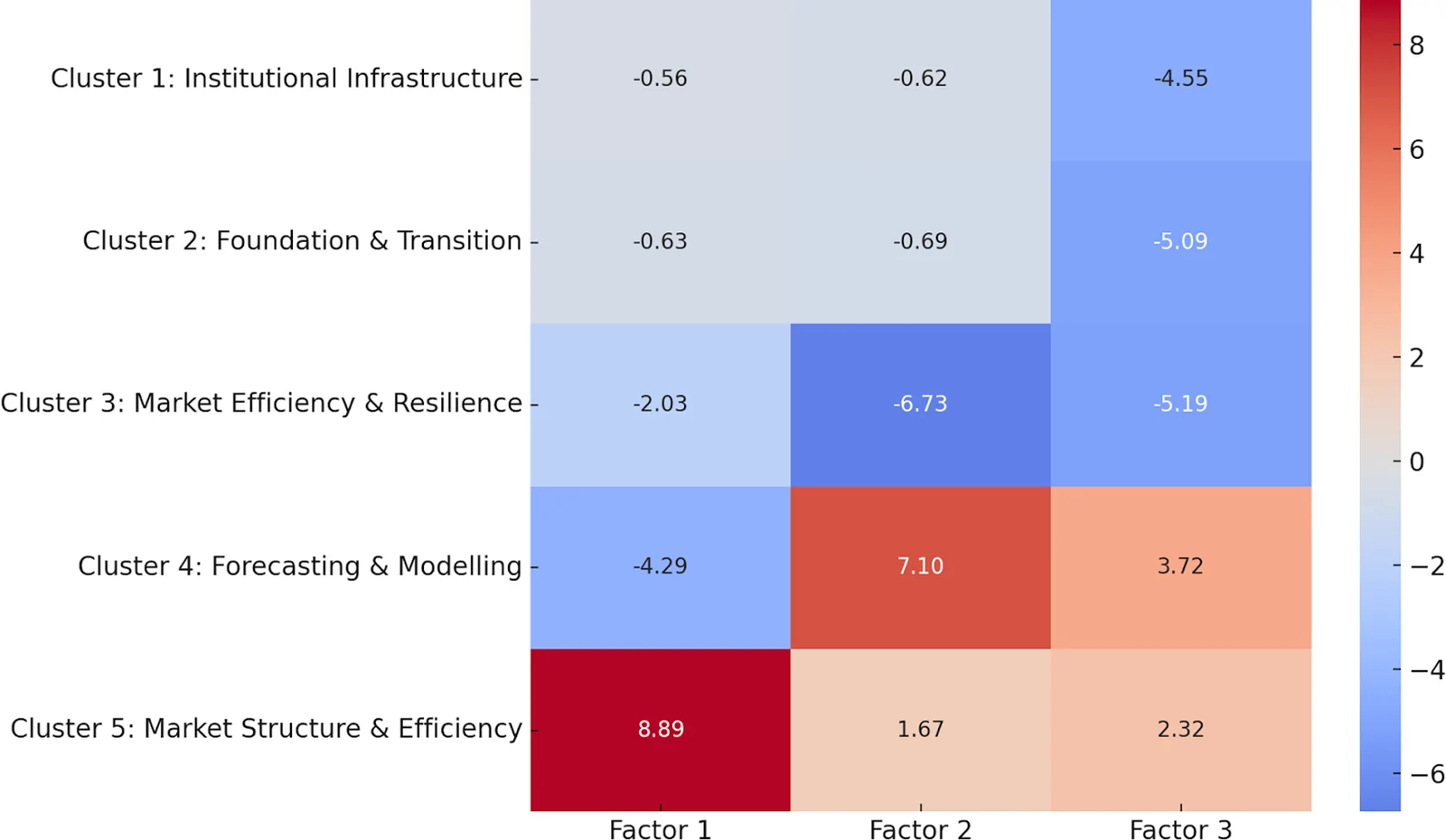
Heatmap of factor loadings by cluster. [Source: Author’s own work].

Uncertainty about the appropriate mechanism of cryptocurrencies introduces a significant challenge for both academia and industry. This study intends to examine variables of cryptocurrency market microstructure by applying methodology incorporating podcast analysis. The study further aims to cluster the identified variables using lexicometry techniques and subsequently factor them. Podcasts featuring expert interviews with finance industry professionals are used. The results of this study lay the foundation for future research to estimate these factors for a thorough understanding of cryptocurrency market microstructure. Additionally, it sets the foundation for developing frameworks based on the results of the study.

## 2. Background

### 2.1 Literature background

Market Microstructure has emerged as a branch of financial economics dealing with extensive understanding of asset price formation. It examines various determinants, such as, trading process, liquidity, regulatory governance and much more. The concept of market microstructure was originally formulated by Garman (1976) in his research on order flow and market maker behavior. Later, theoretical models along with empirical analysis were incorporated to understand various aspects of trading cost, bid-ask-spread, market efficiency etc ( Easley and O’Hara 1987; O’Hara 1998).

In more recent developments, emergence of technology has resulted in research on electronic trading platforms. Authors ( Hasbrouck 2018; Biais et al. 2005) examined the impact of algorithmic trading and high-frequency trading respectively, on market quality, liquidity and volatility. Additionally, research has focused on fragmented markets ( Kohli 2014; Bastidon and Jawadi 2024), dark pools ( Kratz and Schöneborn 2015) and the impact of regulations on markets ( Cumming et al. 2019). Authors ( Easley et al. 2021) have studied machine learning and artificial intelligence techniques impacting microstructural fundamentals.

Developments in microstructure research include the role of decentralized finance and the impact of geopolitical risks on market stability ( Theiri 2024). The microstructure research continues to evolve with the advancements in market surveillance and regulatory technology, which maintain the market integrity in increasingly sophisticated trading mechanisms.

*Traditional market microstructure*

Traditional markets are composed of different channels of investment which vary in features, risks, returns, and mechanism. It includes assets including equities, bonds, commodities, and foreign exchange. For research in microstructure, equities have been widely studied with research in a wide range of areas including, role of market makers ( Tripathi and Dixit 2020); price discovery ( Schwartz et al. 2022); design of trading platform ( Francioni et al. 2008) and much more. Recent studies are examining the impact of exchange- traded funds ( Atilgan et al. 2022) and passive investing on price discovery ( Ben-David et al. 2025).

Bond market, on the other hand, has emerging studies due to post-crisis regulatory changes that shifted trading from over-the-counter (OTC) to electronic platforms ( Bessembinder et al. 2023). Additionally, the foreign exchange market has seen an increase in the number of studies focusing on order flow heterogeneity and role of bank interventions ( Goyal 2016). Commodities markets also reveal unique microstructure characteristics due to physical delivery constraints and speculative trading. Research by Cheng and Xiong (2014) explores how financialization affects commodity price dynamics. Additionally, derivatives markets have been analyzed for their price discovery mechanisms and liquidity spillovers ( Wang et al. 2025).

*Cryptocurrency market microstructure*

Cryptocurrencies emerge as a unique asset class due to their decentralized nature and lack of traditional intermediaries. Early studies highlighted Bitcoin’s price volatility and liquidity dynamics ( Takaishi and Adachi 2020); market efficiency ( Le Tran and Leirvik 2019); and convergence between cryptocurrencies over time ( Apergis et al. 2021). Subsequent research has examined order book behavior, market manipulation, and the role of cryptocurrency exchanges ( Hautsch et al. 2024).

A major distinction in cryptocurrency markets is the prevalence of retail investors and algorithmic trading bots, which contribute to extreme volatility and flash crashes. Research by Tripathi and Dixit (2020) reveal that liquidity pools in digital markets comprise different microstructure properties than that of traditional financial markets. The emergence of stablecoins have also introduced new microstructure dynamics, as they serve as both trading pairs and liquidity providers ( Catalini et al. 2022). To summarise, despite the growth of research in cryptocurrency space, challenges remain in assessing the market due to several factors that remain underexplored. For instance, fragmented markets, social media sentiments, noise trading, and regulatory uncertainty posit as a challenge to determine the actual market value.

### 2.2 Theoretical background

The traditional microstructure theory has primarily provided two types of price-setting behaviors, namely inventory models and information asymmetry models. The focus of inventory models has been on the role of liquidity providers, majorly market makers who adjust their inventory in response to trade flows and price fluctuations. The inventory-based approach originates from Garman (1976) who emphasized the role of market makers in maintaining equilibrium between buy and sell orders. This implies that market makers must actively adjust prices concerning the inventory. Building on this, Amihud and Mendelson (1986) present a similar framework by emphasizing on market-makers pricing problems, suggesting a compensation for holding risky positions. Ho and Stoll (1983) extended this idea by focusing on risk-averse dealers’ inventory, highlighting how inventory imbalance may lead to price adjustment and restored equilibrium. Roll (1984) further suggested a model for high-frequency trade prices to estimate the bid-ask spread rather than reflecting actual changes in the asset’s value.

Later, Stoll (1989) expanded the literature by empirically testing the relationship of bid-ask spread with trading volume and market liquidity. Hasbrouck (1988) contributed to it by estimating the impact inventory balances have on price formation and changes. Madhavan and Smidt (1993) highlighted the nature of desired inventory level, focusing on the outcomes of these inventory models that market makers adjust in response to order flow, which in turn impacts price volatility.

Moving forward, Huang and Stoll (1997) proposed a two-way and three-way model advancing the understanding of inventory risk influence on bid-ask spread. The two-way model does not separate adverse selection and inventory holding components, whereas in the three-way model adverse selection, order processing, and inventory holding are allowed for identification. The work of Bollen et al. (2004) explored the role of inventory management in options markets where it is required to balance inventory by the market makers.

Asymmetric information-based models explain the market behaviors and how information disparity among investors affects the market prices. Copeland and Galai (1983) explored the phenomenon of showing the effect of bid-ask spread on risk of trading with informed investors. Further, Glosten and Milgrom (1985) expanded the study by highlighting adverse selection costs for liquidity providers.

Easley and O’Hara (1987) expanded the previous framework by comparing between informed and uninformed traders. Informed traders strategically trade at times when they can earn maximum profit, while uninformed traders are engaged in random activities leading to price adjustment. O’Hara (2003) offered a comprehensive view of how uninformed traders disrupt market activities leading to information asymmetry. Consequently, Easley et al. (2002) used the Probability of Informed Trading (PIN) model to examine the relationship between information asymmetry and asset returns. O’Hara and Ye (2011) expanded on these models by examining the role of algorithmic trading with information asymmetry, providing a modern perspective on the evolution of market dynamics.

## 3. Data and methods

### 3.1 Analysis database

This study is drawing on Eisenhardt et al. (2016) inductive-theory building methods with a focus towards an exploratory approach to discover the true inner meanings of market microstructure and produce an improved comprehension of how it can be applied to the cryptocurrency market. The sample selection is based on the approach employed by Fisher et al. (2020), in which podcasts featuring expert interviews of finance professionals are used as the data source. Podcasts selected for this study are interviews with the leading industry resources in financial markets to create improved understanding and outline the market microstructure of the cryptocurrency market. Table 1 lists the podcasts that were included in the study.

**Table 1.** Podcast list.

S.No.	Podcast	Speaker	Designation	Company	Date
1	Why The Short Volatility Trade Is Back And Bigger Than Ever | Odd Lots	Kris Sidial	Co-CIO	Ambrus Group	2024
2	Ai Who? It’s All About Market Microstructure With Shelley Eleby Of Bmo Capital Markets	Shelley Eleby	Managing Director And Head Of Marketing For Electronic Trading	BMO Capital Markets	2023
3	Where Market Making Meets Market Microstructure	Sasha Stoikov	Senior Research Associate	Cornell Financial Engineering	2023
4	Field Testing Changes In Market Microstructure	Rob Gouley & Eric Stockland	Trader & Managing Director	OMERS & BMO Capital Markets	2021
5	Understanding Crypto Market Microstructure (W/ Coindesk Head Of Research, Noelle Acheson)	Noelle Acheson	Head Of Research	Coindesk	2019
6	Market Microstructure, Hfts, And Iex	Eric Hunsader	CEO & Hedge Fund Manager	Nanex	2016
7	Understanding Market Crashes (Market Microstructure Overview	Albert S. (Pete) Kyle	Charles E. Smith Chair Professor Of Finance	University Of Maryland's Robert H. Smith School Of Business	2020
8	Focus On Market Microstructure: Venue Footprint Analysis	Linda Giordano/ Jeffrey Alexander	Founder/ Senior Analyst	Babelfish Analytics/ Brigadier Capital	2013
9	Wei Dai: Fighting For Every Basis Point	Wei Dai	Head Of Investment Research And A Vice President	Dimensional Fund Advisors	2024
10	I Enjoy Trying To Win A Marathon Rather Than Winning Sprints	Eric Crittenden	CIO	Standpoint Asset Management	2020
11	How Decentralized Is Bitcoin Mining W/ Harry Sudock	Harry Sudock	Bitcoin /Mining Expert	CleanSpark	2023
12	What Will Crypto’s Market Structure Look Like?	Alex Gordon Brander	Founder	Omega One	2019

[Source: Author’s own work].

The podcasts considered for this research provide a multi-dimensional view of the cryptocurrency and traditional finance industries ranging from academia, trading, and research to executive leadership. Furthermore, professionals such as Sasha Stoikov and Shelley Eleby possess expertise in both traditional as well as digital markets, making it possible to have comparative views regarding the ways microstructure influences the decentralized investment mechanism. The study examines the publicaly available podcasts, focusing on episodes where the experts discuss specifically about market microstructure. The major aim of short-listing podcasts was to prioritize certain segments wherein experts describe empirical observations, technological interventions, and traditional versus decentralized markets.

In order to ensure coverage of both theoretical frameworks and operational realities, a podcast from an academic researcher was also included for the study. Additionally, the transcripts of the study were firstly, manually analyzed to bridge practitioner expertise with academic inquiry, in order to offer perspectives on cryptocurrency market microstructure’s evolution. For instance, Wei Dai’s (Dimensional Fund Advisors) emphasis on “fighting every basis point” aligns with microstructure theory’s focus on transaction cost minimization, while Alex Gordon-Brander’s (Omega One) commentary on cryptocurrency’s market structure highlights gaps in regulatory alignment. The objectives of adopting this approach were diversity of expertise by combining insights from varied experts, real-world validation of theoretical assumptions through practitioner insights, and gap identification.

### 3.2 Analysis process

The study employed a structured approach ( Figure 1) to analyze insights from cryptocurrency market microstructure experts featured in publicly available podcasts. The podcast episodes were transcribed using TurboScribe, an automated transcription tool, followed by manual verification to ensure accuracy in financial terminologies and speaker contribution. The transcripts were manually and systematically reviewed to extract the relevant content, focusing on the discussions related to the study. The information obtained was consolidated into a single corpus for descending hierarchical classification (DHC) using open-source software Iramuteq ( Ratinaud 2014). Following the completion of DHC, the present study proceeded with the application of factorial analysis.

For the purpose of DHC, processes developed by Chaney and Séraphin (2023) were employed for processing large corpus of textual data and drawing inferences from it. Stepwise procedure outlined by Gourlay (2019) was followed for the generation of input text corpus, which was later imported to Iramuteq for processing. By integrating manual content extraction with computational text analysis, the study bridges qualitative expertise with quantitative confirmation, providing an integrated view of evolving microstructure of cryptocurrency markets.

The adoption of descending hierarchical classification uncovers its capacity to systematically detect latent structures and relationship structures within textual data. DHC enables the division of large corpus of data into logically themed clusters by extracting statistically important co-occurring themes. Segmenting corpus by DHC, provides an empirical basis to identify the dominant themes without relying on assumptions, ensuring a purely quantitative exploration.

Subsequently, factorial analysis was adopted to examine the interrelation of variables and reveal the hidden structures that shape the organization of themes across the vast research landscape. The aim of employing factorial analysis is to minimize the dimensionality of complex datasets without sacrificing meaningful variations. While DHC categorizes variables in terms of lexical similarity and frequency patterns, factorial analysis acts as a complementary perspective to refine these variables, making the interpretive insights derived from DHC more significant. This analysis helps in enhancing the explanation capability of analysis by noting down the most effective factorial space.

For the analysis, a set of keywords was selected, focusing on those with frequency percentage of more than 10% to ensure the inclusion of variables that meaningfully contribute to the cluster’s thematic identity. In order to avoid statistical noise, low-frequency keywords (below 10%) were excluded from the analysis. Further, a variable matrix was constructed, capturing the co-occurrence and frequency of keywords. The analysis was performed using Python 3.10 with a scikit-learn library to implement the factor analysis model. It was selected for its reliability in handling multivariate statistical procedures and its superior capacity to handle large datasets.

## 4. Findings

Following are some sample quotations from the podcasts:

*“And we both agree that there’s 100 percent of market microstructure impact. And we have no idea how to quantify how big it actually is. Because you see, you know, you see a sailor wake up in the morning or an ETF wake up in the morning. They might buy a month’s worth of net new issuance. Or a year’s worth of net new issuance, as the case may be, as we get further and further out the epochs.”*

*- Harry Sudock, Clean Spark, 2023*

*“When you think about high frequency trading and traditional markets, you’re talking about millions, tens of millions of dollars of investment to create microsecond improvements and connectivity to exchanges. Most exchanges in the crypto world, however, are based on like web based, which means there’s no way they can actually support that kind of reduce the agency in the first place. And in large, but that actually makes it more democratic, right? It’s this idea of being able to trade.”*

*- Noelle Acheson, CoinDesk, 2019*

*“Initially crypto and bitcoin, but we also have our eye on the future where we see stock spawns, real estate and currencies all moving over into this digital environment.”*

*- Alex Gordon Brander, Omega One, 2019*

### 4.1 Descriptive statistics

**Figure 2.**
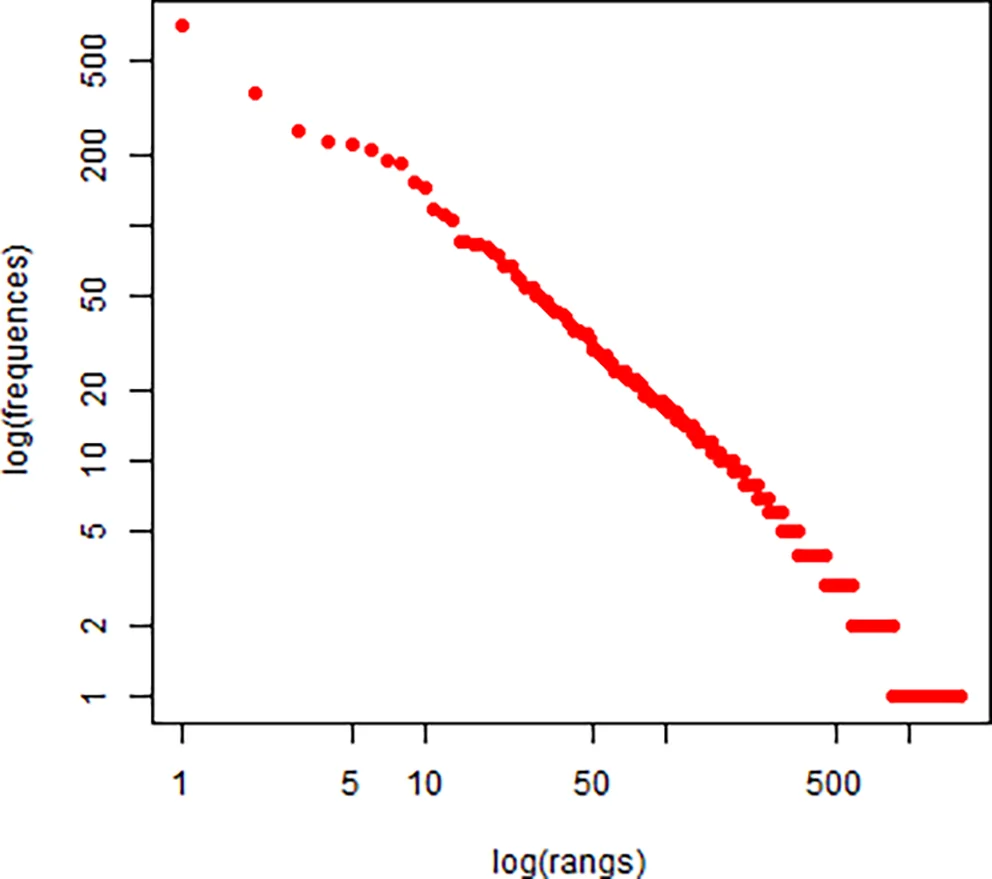
Zipf diagram of descriptive statistics. [Source: Author’s own work].

Figure 2 represents log-frequency distribution of lexical items in the corpus that is plotted against the rank-ordered term frequencies of log-scale. The x-axis denotes term ranks, 1 being the most frequent, while the y-axis represents log-transformed raw frequencies. Key reference values (1-500) are marked on the y-axis to highlight exponential decay in the term usage. The near-linear slope confirms Zipf’s law, with high-frequency terms dominating the distribution.

The downward slope of the graph highlights the classical distribution pattern. The top five high-frequency terms that were identified from analysis were market (182 occurrences), price (110 occurrences), trading (86 occurrences), stock (44 occurrences), and cryptocurrency (43 occurrences). On the other hand, the rare words that were identified were: recession (2 occurrences), profitability (2 occurrences), outperform (2 occurrences), infrastructure (2 occurrences), and fragmentation (1 occurrence). This analysis acts as a base for further exploration of themes formed in the analysis.

### 4.2 Descending hierarchical classification

**Figure 3.**
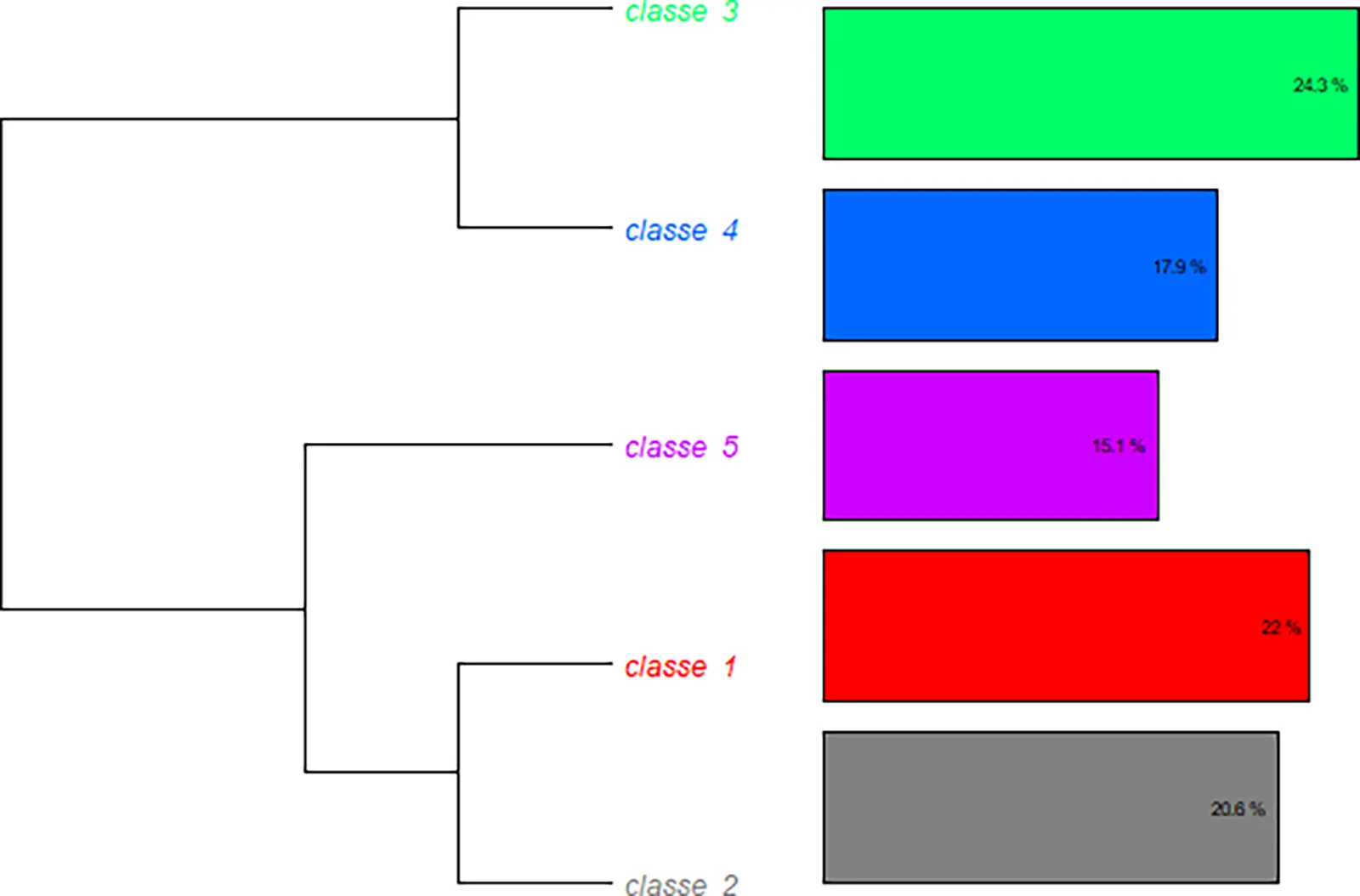
Descending hierarchical classification. [Source: Author’s own work].

**Figure 4.**
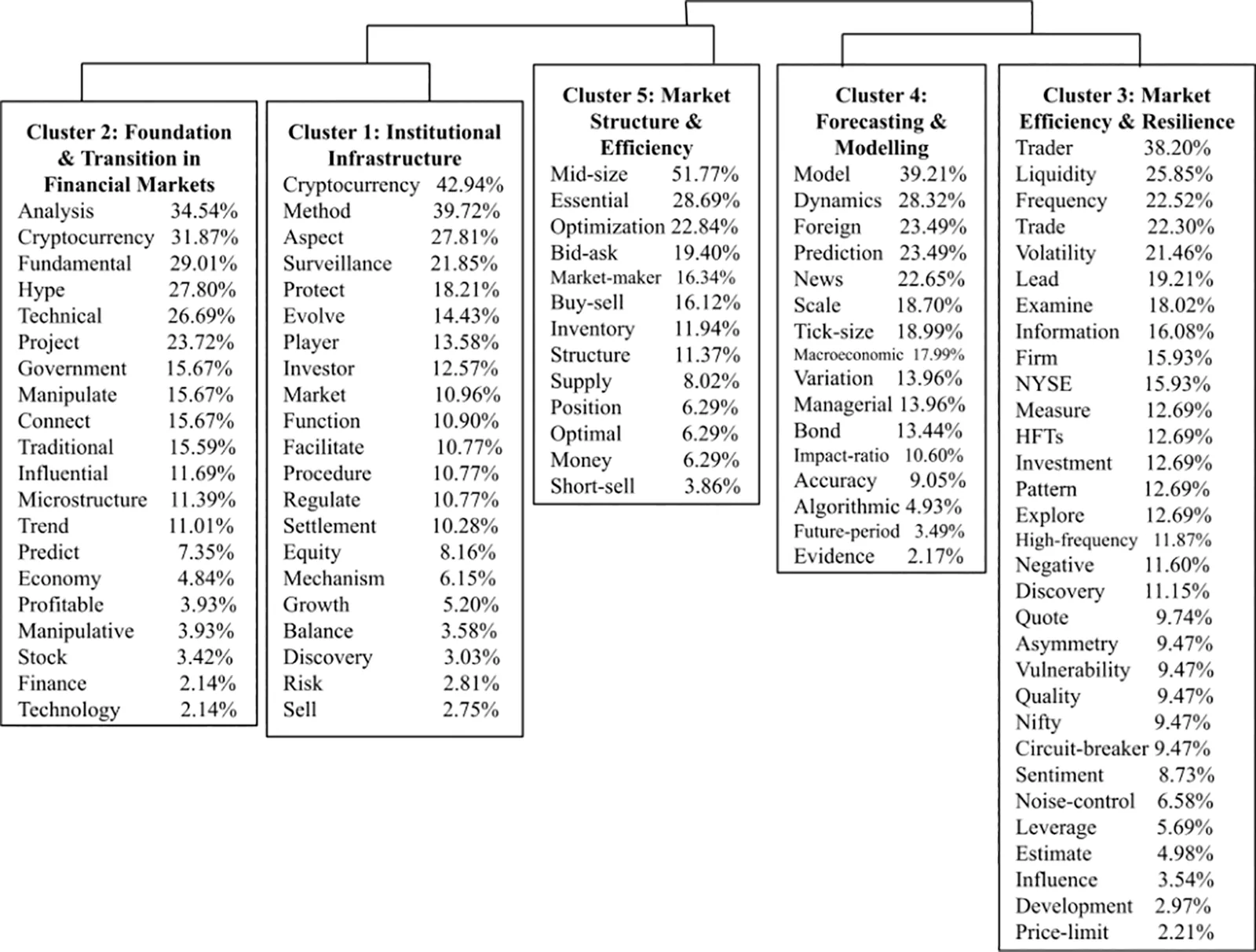
Clusters of DHC. [Source: Author’s own work].

Table 2 represents a statistical summary of the DHC results derived from the transcription database. The corpus under analysis contains a total of 7,265 word occurrences, representing 1,625 distinct forms. It indicates the presence of repeated usage of many words. Among these, 774 are the words that appear only once in the entire corpus, highlighting the lexical diversity of the text. The DHC further reveals 5 clusters ( Figure 3), each representing a unique thematic area within the experts’ discussion on market microstructure. The classification of keywords into clusters ( Figure 4) provides insights into distinct conceptual structures used by financial market experts.

**Table 2.** Summary of descending hierarchical classification.

No. of occurrences	No. of forms	No. of hapex	Mean of occurrences by text	No. of text segments	No. of clusters
7265	1625	774	3421.67	918	5

[Source: Author’s own work]

**Cluster 1: Institutional Infrastructure**

The cluster focuses on regulatory, procedural and institutional frameworks that support the functioning of financial markets. The prominent role of governance in market microstructure is reflected upon, wherein exchanges, regulators, and market participants engage in order to ensure transparency and fairness along with maintaining stability. To look from an academic perspective, this cluster aligns closely with the market design theory ( Roth and Wilson 2019), which clearly states that the structural rules significantly influence the behavior of participants resulting in an impact on the market outcomes. The cluster suggests a technical discussion around the process of buy and sell orders in the marketplace. It also reflects on the ability of investors to identify risk control frameworks and take the best available opportunity throughout the range of different trading venues. These factors revolve around the microstructure models that incorporate institutional frictions such as the Glosten and Milgrom (1985) model on informed trading and pricing.

Additionally, the mention of cryptocurrency in this regulatory context enhances concerns about regulatory arbitrage, cross-border risk transmission, and technology-driven disintermediation. These interpretations necessitate examination of adaptive regulatory frameworks that can facilitate a variety of financial instruments along with strong systemic protection. Additionally, investor protection and risk aversion concerns refer to an intersection of market microstructure with law and economics in this cluster. The regulatory concerns are emphasized to act as fundamental pillars of market functioning in this cluster.

**Cluster 2: Foundation and Transitions in Financial Markets**

The cluster reflects an underlying tension between traditional finance and continuous emergence of decentralized digital markets, most notably cryptocurrencies. Dominance of leading terms like “fundamental”, “technical”, and “project”, alongside cryptocurrencies present challenges in applying traditional valuation techniques in drastically volatile and innovation-driven markets, such as blockchain technologies and digital tokens. This cluster is majorly concerned with industry issues of applying traditional tools, such as, ratio analysis, trend analysis, or project feasibility methods for risk and returns associated with digital assets. These tools work well for analyzing stable and predictable environments ( Adamyk et al. 2025), but with the peculiar problems in cryptocurrencies, it is concerning that traditional models will provide accurate results. Experts have critically discussed exploring and adapting valuation frameworks for improved estimation of emerging markets.

The other emphasis of this cluster reflects concerns about information asymmetry, market speculation, and external control as the most essential issues in microstructure theory. Researchers have stated that markets can never become informationally efficient as a result of the expense of information acquisition ( Grossman and Stiglitz 1980; Asparouhova et al. 2024). These expert interpretations raise concerns on the ability of traditional microstructure theory to explain the behavior of digital assets, where non-fundamental sentiments and social-media hype are the core dominant price action.

**Cluster 3: Market Efficiency and Resilience**

This cluster identifies the fast-evolving and technologically intensive features of market microstructure. The intensity in high-frequency trading, market volatility, and behavioral signals by experts is identified in this cluster. The occurrence of words “frequency”, “HFTs”, and “noise-control” indicate the emphasis on algorithms, arbitrage and liquidity features. These expert views are consistent with the literature on speed-based competition and latency-induced segmentation of markets ( Budish et al. 2015). Other dominant words (sentiment, volatility, asymmetry) indicate the role played by behavioral and informational factors. This reflects on the interlinkage between microstructure noise models ( Bacry et al. 2013) and behavioral finance, where decision-making based on emotions, behavioral biases, and information overload affect the market stability.

Furthermore, the cluster also highlights the inter-market comparison (NYSE, Nifty, circuit-breakers, noise-control) implying a comparative analysis of regulatory tools used to manage market instability. Circuit-breakers, in particular, reflect on the response of institutions to technological disruptions and serve as key tools in maintaining the market during speculative bubbles. On the other hand, the integration of “NYSE” and “Nifty” with other terms in the cluster (asymmetry, fairness, efficiency) indicate a central concern with structural equity in financial markets. These terms reflect the idea that not all markets operate equally, and some may offer unequal opportunities to investors due to the inherent design of informational imbalance.

In addition to this, the cluster also suggests concerns about access inequality (difference, asymmetry), wherein institutional investors with algorithmic access, better data, and cross-border capabilities can leverage arbitrage opportunities, enhancing price discovery. This raise concerns in the field of market microstructure on how modern financial markets function and the challenges they carry along with of transparency, fairness and efficiency. These dynamics suggest a need for academic research into regulatory solutions and deployment of technologies that promote equal access and reduce disparities between markets.

To conclude, this is the largest cluster formed which is inclusive of mixed interpretations of expert insights. It effectively bridges technological determinism with behavioral market theory, portraying a market landscape where speed, sentiment and surveillance interact to determine efficiency and resilience. It also raises concerns on information gaps for institutional and retail traders, inviting further research into regulatory and technology equalization. These aspects may act as potential solutions for minimizing inter-market distortions.

**Cluster 4: Forecasting and Modelling**

The experts have highlighted the analytical modeling and predictive aspects of market microstructure that are reflected in this cluster. The prominent terms (model, dynamics, prediction) suggest a strong orientation towards the theoretical and quantitative aspects that replicate and forecast market behavior. The managerial and macroeconomic aspects highlighted in the cluster significantly highlight the emphasis on incorporating broader economic variables and decision-making frameworks into the traditional models of microstructure. It emphasises on the evolution of market microstructure roles beyond pure trade mechanics towards a wide understanding of market behavior ( Bouchaud 2022).

Additionally, less frequent terms (algorithmic 4.93%) urge the role of computational finance and automation, suggesting an increasing adoption and reliance on machine learning, agent-based models, and high-frequency trading. Other low frequent terms (future-period, evidence) seem to insist on powerful empirical grounding in addition to the theoretical contributions. To conclude, the cluster represents a mix of methodological approaches for microstructure studies. It majorly focuses on technically advanced and data-driven approaches for macroeconomic indicators, market behavior and predictive analytics.

**Cluster 5: Market Structure and Efficiency**

The formation of cluster 5 consolidates the expert insights on the operational microstructure of markets, delving into the true inner meanings of price formation, and how liquidity is maintained. The core dominant terms (bid-ask, inventory, market-maker, buy-sell) in this cluster firmly situate it with the inventory-based models of market microstructure, such as the Ho and Stoll (1981), wherein dealers adjust prices based on inventory imbalances and adverse selection risks.

Other key terms (mid-size, optimization) suggest a particular focus of the experts on less liquid or developing markets. In such scenarios, structural inefficiencies and information asymmetries are more prominent wherein market participants face challenges in balancing the inventory costs and spread management. Additionally, the occurrence of “short-sell” in this cluster can have a two-faced interpretation. Short-selling is often viewed as a mechanism for correcting over-valuations in the market, enhancing price discovery ( Long et al. 2024). However, other scholars bring concerns of market destabilization and manipulation due to short-selling ( Jung et al. 2013).

To interpret from a theoretical viewpoint, this cluster majorly focuses on the role of microstructure in affecting macro outcomes such as volatility, price informativeness, and investor behavior. It reflects on the expert-level understanding of how micro-level trading environments affect macro-level market performance.

### 4.3 Factorial analysis

The factorial analysis revealed patterns based on the clusters formed through DHC. A total of three factors were formed based on the analysis. Figure 5 presents the factor loadings for the 5 thematic clusters. The loadings (-6 to 8) indicate the strength and direction of the association between each cluster and latent factors.

Factor 1 emerges as the most dominant and primarily inclusive of cluster 5 of the DHC. It suggests that the first factor primarily captures operational and structural aspects of the cryptocurrency market, such as bid-ask, buy-sell, market-maker. These aspects are foundational in understanding the thematic organization of cryptocurrency market microstructure. Factor 2 is characterized by a strong positive association with cluster 4 and a negative association with cluster 3. It indicates that this factor contrasts predictive analytics with resilience mechanisms. While the forecasting techniques are prominent, they operate independently of the efficiency and resilience aspects such as volatility, liquidity, and high-frequency trading.

Further, factor 3 presents a divide between the clusters. It is positively influenced by cluster 3 and 4, whereas negatively related to cluster 1 and 2. This combination reflects a contrast between dynamic market efficiency and foundational themes. The factor points to a shift in thematic emphasis from foundational constructs towards dynamic market performance.

The application of factorial analysis has refined the complexities involved in interpreting podcast data into explicit 3 factors, providing theoretical as well as practical insights. The results of this analysis highlight how market structure, forecasting models, and resilience mechanisms shape the currency understanding of cryptocurrency market microstructure. Table 3 gives a thematic overview of how each latent factor structures the relationship between each factor. Factor 1 emerges as a backbone of operational market dynamics, emphasizing the core mechanics behind core mechanisms behind crypto market functioning. Factor 2 reveals the analytical tension between forward-looking modelling and the market’s inherent efficiency under variable conditions. Finally, factor 3 signals a structural divide between old and new market priorities, highlighting a shift away from institutional setups towards dynamic, adaptive and market performance.

**Table 3.** Thematic map of clusters.

Factor	Dominant Clusters
Factor 1	Cluster 5 (largely positive loading)
Factor 2	Cluster 4 (positive), Cluster 3 (negative)
Factor 3	Cluster 3, 4 (positive), Cluster 1, 2 (negative)

[Source: Author’s own work]

## 5. Discussion

The findings of the study provide crucial insights into the complex dynamics shaping the financial markets. The extracted clusters through DHC analysis form an integrated picture of the complex understanding of cryptocurrency market microstructure, driving academic innovation and practical implications within the financial industry. The institutional infrastructure cluster underlines the significance of digital asset governance and market oversight. The rise of decentralized finance or DeFi has emerged with new regulatory challenges for maintaining market integrity and investor protection ( Shukla et al. 2024). To illustrate, organizations such as the European MiCA (Markets in Crypto-Assets) framework ( Conlon et al. 2024) and the Securities Exchange Commission’s evolving stance on digital assets classification in the United States ( Guseva 2020) are some examples of the regulatory efforts worldwide.

Further, the next cluster, foundation and transition clearly reflect the evolution of financial markets from traditional setting to a hybrid or digital structure. The cluster reflects a challenge in adopting traditional financial theories for the cryptocurrency setting due to factors such as speculative behavior, social media hype, and technological disruptions. This highlights a need for institutional as well as retail investors to navigate the transitions wisely, balancing log-held valuation models with new age sentiment and momentum effects ( Mikhaylov et al. 2023). The popular GameStop and AMC “meme stock” scenarios clearly explain how the microstructure fundamentals were disrupted by investor behavior, reflecting an unreasonable and manipulated market. The next cluster, that is, market efficiency and resilience, deepen the ability of markets to absorb shocks, maintain liquidity, and recover from volatile events. The rise of high-frequency and algorithmic trading in recent years has a major influence on the market conditions. It has opened possibilities for enhanced liquidity provisions ( Hendershott et al. 2011) on one hand, whereas, on the other hand, it introduces new system-related vulnerabilities, such as the flash crashes ( Akansu 2017). They focus on regulatory and technological measures towards ensuring a level-paying market.

The fourth cluster, forecasting and modelling, highlights the central role of predictive analytics and quantitative modelling in the creation of today’s financial markets. Organizations are relying more on machine learning and AI-models to enhance forecasting accuracy ( Rachakatla et al. 2023). These models are well integrated with factors such as, social media sentiment, web scraping, and much more, for better precision in the predictions ( Lv et al. 2022). For instance, asset management companies like the Quant Mutual Fund in India, or hedge funds like Two Sigma in the United States, heavily rely on quantitative techniques that not only process traditional financial indicators but also integrate real-time data to anticipate price fluctuations and market trends. However, the cluster also captures the risks and limits involved due to over-reliance on models. The 2007-2008 financial crisis exposed how risk estimation models underestimated the probability and impact of events such as the black swan ( Walter 2020). While forecasting models are crucial tools for portfolio management and risk assessment, their adaptability remains a critical challenge.

Finally, the last cluster, market structure and efficiency reflect on the operational mechanisms that support market liquidity and transaction costs. The role of market makers is crucial to ensuring a smooth order execution, especially in fragmented markets like the cryptocurrency market, where trading occurs across dozens of exchanges ( Galati 2024). Moreover, the inclusion of short-selling and price-formation act as an additional question on the market fairness, efficiency and integrity. The European sovereign bond markets during the Eurozone debt crises (2010-2012) depleted completely as investors rushed to sell the distressed sovereign bonds causing bid-ask-spread to widen largely. Additionally, the GameStop short squeeze in 2021 highlighted how investors, through social media influence, disrupted the market mechanism. Thus, market structure is constantly shaped by institutional behavior, technology, and much more, making it crucial for both industry as well as academia.

## 6. Implications

**Academic Implications:** The five clusters that were formed through descending hierarchical classification of podcast transcripts of finance industry experts, acts as a base for future academic exploration of cryptocurrency market microstructure. By concentrating the scattered variables into meaningful and related clusters, the study provides a roadmap for empirical and theoretical development, particularly in the area of digital financial markets.

**Practical Implications:** For the industry insights, the findings of the study provide actionable insights into key drivers and vulnerabilities. Practitioners can leverage the identified variables to inform strategy formulation, in terms of regulatory compliance, liquidity management and predictive analytics. For instance, understanding the complex interactions between liquidity provisions, volatility, and circuit breakers can help market participants better navigate periods of systemic stress. Similarly the emphasis on forecasting and modeling deepens the importance of adaptive analytics that can incorporate emerging data sources and account for changing market dynamics.

## 7. Limitations and future research agenda

While the study adopts unique techniques of podcast analysis in integration with lexicometry and factorial analysis, however it has some limitations. However, it provides potential future research agendas in the subject area of cryptocurrency market microstructure. The major areas for future research directions are as follows:

i) Incorporating advanced dimensionality reduction techniques: The study focused on the factorial analysis based on the results of descending hierarchical classification. Future research can adopt methods like principal component analysis (PCA) or Exploratory Factor Analysis (EFA) to reduce multicollinearity, validate factor loadings, and extract latent dimensions from large datasets. Future research questions can be as follows:
FRQ1: Does the integration of PCA-based components improve the predictive power of models forecasting price efficiency?
FRQ2: How strong are the factor structures across different time periods, markets, or asset classes when subjected to advanced dimensionality reducing techniques?
ii) Empirical validation through large-scale datasets: The current study relies on podcast transcripts and keyword frequencies, providing qualitative insights through top industry experts. The future studies can expand the research by including large-scale transaction dataset for quantitative analysis and empirically test the identified relationships. Future research questions can be as follows:
FRQ3: How do empirically observed quantitative datasets align with the clusters and factors derived from qualitative data?
FRQ4: Does combining qualitative and quantitative datasets yield a better understanding and exploration of cryptocurrency market microstructure?
iii) Technology disruptions and adaptive market structures: The rapid evolution of blockchain technology, automated market-making (AMM) and DeFi is reshaping the traditional assumptions of market structure. Future research should explore how these innovations challenge the existing theories of financial markets, particularly market microstructure. Future research questions can be as follows:
FRQ5: Do technological upgrades alter the traditional market microstructure characteristics?
FRQ6: What are the long-term impacts of AMM on blockchain based assets, particularly cryptocurrencies?
iv) Regulatory interventions and market quality: Given the prevailing global regulatory attention on cryptocurrency markets, especially concerning investor protection and systemic stability, there is an urgent need to assess how regulatory shifts affect the market quality and participant behavior. Future research can bridge empirical evidence with regulatory design to inform a balanced, effective policy framework.
FRQ7: How do regulatory interventions influence price formation in the cryptocurrency market?
FRQ8: How can the regulatory framework be designed to balance innovation incentives with the need for transparency and market integrity?

## 8. Conclusion

The rapid advancements in the cryptocurrency market present significant challenges for retail as well as institutional investors to understand its mechanism for informed decisions. Market microstructure of cryptocurrencies, hereby, remain an underexplored area despite the growth and increasing integration of digital assets into the global financial systems. Exploring the dynamics of cryptocurrencies through industry insights, this research aimed to identify and classify the key variables that shape the structure of cryptocurrency markets. To achieve this, the study adopted a mixed-methodology approach. A qualitative method of podcast analysis was adopted to explore industry insights on cryptocurrency market microstructure. These insights were quantitatively explored through techniques of lexicometric analysis using descending hierarchical classification which revealed 5 cluster formation. Further, the factorization of variables was done using Python 3.10. As a result, this study contributes to the growing body of knowledge on cryptocurrency market microstructure by bridging industry insights with academic understanding. It lays groundwork for further empirical research and regulatory contributions in this rapidly evolving domain.

## Ethical statement

1. Ethical approval is not required because the study is based on podcast analysis of industry experts without any direct involvement.
2. There is no direct involvement of human participants as it includes interviews of industry experts through the medium of podcasts.
3. All the details of the methods used in the study have been thoroughly explained in the manuscript.
4. The article does not contain any data that has to be made publicly available. All the data and values are included in the manuscript only.

## Data Availability

No data associated with this article.
