# Supplementary material for: Decoding Market Voices: Beyond the Charts Analysis of Cryptocurrency Market Microstructure
Source: F1000Res. 2026 Jun 23;15:274. Originally published 2026 Feb 16. [Version 2] doi: 10.12688/f1000research.173888.2 (PMC13550928; doi:10.12688/f1000research.173888.2)
Supplement: Peer review history

# Reviewer report: Reviewer response for version 2

**DOI:** 10.5256/f1000research.203003.r497351

**Published:** 2026-08-03

**Authors:** Jayati Ranga

**Affiliations:** 1 Amity University, Punjab, India

The progression of the paper is well articulated but in section like Discussion, across clusters repetitive explanation is observed also the qualitative cryptocurrency reserach need to be highlighted more. The mixed-method approach combining podcast analysis, lexicometric analysis using IRaMuTeQ, and factor analysis is innovative and appropriate for the exploratory objectives. But the selection of 12 podcasts could have been expanded further despite indicating thematic saturation.

 Following areas could have been added:

- IRaMuTeQ parameter settings.
- Complete preprocessing workflow.
- Version details of all software packages.
- Factor analysis parameters (rotation method, extraction method, convergence criteria).
- Statistical reporting could have been improved by including KMO, Bartlett's Test, Eigen value, Percentage variance, Communalities, Factor loading cut off values, reliability of extracted dimensions.

Though the podcast episodes are publicly available, but the transcripts could have been added, along with python scripts, IRaMuTeQ project file.

 Future research directions could have been emphasized in order to provide empirical validation using transaction level datasheet.

 The manuscript is good enough with minor improvements.

Is the work clearly and accurately presented and does it cite the current literature?

Yes

If applicable, is the statistical analysis and its interpretation appropriate?

Yes

Are all the source data underlying the results available to ensure full reproducibility?

Yes

Is the study design appropriate and is the work technically sound?

Yes

Are the conclusions drawn adequately supported by the results?

Yes

Are sufficient details of methods and analysis provided to allow replication by others?

Yes

Reviewer Expertise:

International Trade, Human Resource Management, Tourism, Marketing

I confirm that I have read this submission and believe that I have an appropriate level of expertise to confirm that it is of an acceptable scientific standard.

# Reviewer report: Reviewer response for version 2

**DOI:** 10.5256/f1000research.203003.r496030

**Published:** 2026-07-06

**Authors:** Rakesh Kumar Sharma

**Affiliations:** 1 Thapar Institute of Engineering and Technology (Deemed to be University), Indra Puri Colony, Punjab, India

I have carefully examined the revised version of the manuscript along with the authors' point-by-point response to the review comments.

 I am satisfied that the authors have adequately addressed all the comments and suggestions raised during the review process. The revisions have substantially improved the clarity, methodological rigor, presentation, and overall quality of the manuscript. The responses provided are appropriate, and the concerns raised in the previous review have been satisfactorily resolved.

 In my opinion, the manuscript is now suitable for indexing in its present form. Therefore, I recommend **acceptance of the manuscript** without any further revisions.

 I appreciate the authors' sincere efforts in revising the manuscript and addressing the reviewers' observations comprehensively.

**Recommendation:** **Accept for Indexing**

 Sincerely,

Is the work clearly and accurately presented and does it cite the current literature?

Partly

If applicable, is the statistical analysis and its interpretation appropriate?

Yes

Are all the source data underlying the results available to ensure full reproducibility?

Yes

Is the study design appropriate and is the work technically sound?

Partly

Are the conclusions drawn adequately supported by the results?

Partly

Are sufficient details of methods and analysis provided to allow replication by others?

Partly

Reviewer Expertise:

Accounting and Finance and block Chain technology

I confirm that I have read this submission and believe that I have an appropriate level of expertise to confirm that it is of an acceptable scientific standard.

# Reviewer report: Reviewer response for version 1

**DOI:** 10.5256/f1000research.191744.r488866

**Published:** 2026-06-16

**Authors:** Sandeep Paatlan

**Affiliations:** 1 Chandigarh University, Sahibzada Ajit Singh Nagar, Punjab, India

**Summary**

 This manuscript presents an innovative exploration of cryptocurrency market microstructure through the analysis of expert podcast transcripts using lexicometric techniques, Descending Hierarchical Classification (DHC), and factorial analysis. The study identifies five thematic clusters and three latent factors that contribute to understanding cryptocurrency market microstructure from a qualitative perspective. The use of podcast-based expert discourse as a primary data source represents a novel contribution to the literature.

**General Comments**

 The manuscript is well organized, clearly written, and addresses an emerging area of interest within financial markets. The integration of qualitative data with computational text analysis provides a fresh perspective on cryptocurrency market microstructure and offers useful theoretical and practical insights.

 The methodology is generally appropriate and the analytical procedures are described in a logical manner. The findings are adequately linked to existing literature and contribute to the growing discussion on digital financial markets.

 Minor Suggestions

1. The manuscript would benefit from a more detailed justification of the podcast selection process, including clearer criteria regarding inclusion and exclusion of podcast episodes.
2. While the authors describe the transcription and coding process, additional information regarding corpus preparation and validation procedures would further strengthen reproducibility.
3. The study relies on 12 podcasts as its primary dataset. A brief discussion regarding potential sampling limitations and representativeness of expert opinions would improve methodological transparency.
4. The discussion section could further elaborate on how the identified factors may be operationalized in future empirical research using quantitative market data.
5. Although the study introduces a novel qualitative approach, providing access to the processed corpus or keyword extraction framework as supplementary material would enhance transparency and facilitate future replication.

**Conclusion**

 This is a timely and original contribution that introduces an innovative methodological approach to studying cryptocurrency market microstructure. The article is scientifically sound, the conclusions are supported by the results, and the work offers valuable directions for future research. I support indexing following consideration of the minor suggestions outlined above.

Is the work clearly and accurately presented and does it cite the current literature?

Yes

If applicable, is the statistical analysis and its interpretation appropriate?

Yes

Are all the source data underlying the results available to ensure full reproducibility?

Yes

Is the study design appropriate and is the work technically sound?

Yes

Are the conclusions drawn adequately supported by the results?

Yes

Are sufficient details of methods and analysis provided to allow replication by others?

Yes

Reviewer Expertise:

Finance, Financial Markets, Business Management, Quantitative Research Methods, Data Analytics

I confirm that I have read this submission and believe that I have an appropriate level of expertise to confirm that it is of an acceptable scientific standard.

# Reviewer report: Reviewer response for version 1

**DOI:** 10.5256/f1000research.191744.r459534

**Published:** 2026-03-05

**Authors:** Rakesh Kumar Sharma

**Affiliations:** 1 Thapar Institute of Engineering and Technology (Deemed to be University), Indra Puri Colony, Punjab, India

Peer Review Report

 Manuscript Title: Decoding Market Voices: Beyond the Charts Analysis of Cryptocurrency Market Microstructure

 Journal: F1000Research

 1. Overall Assessment

 The manuscript presents a novel and interdisciplinary exploration of cryptocurrency market microstructure by employing podcast transcripts of financial experts as primary qualitative data. The integration of Descending Hierarchical Classification (DHC) using Iramuteq and factorial analysis using Python demonstrates methodological creativity. The study successfully identifies five thematic clusters and three latent factors that structure expert discourse on cryptocurrency market microstructure. The approach is innovative and contributes to qualitative finance research by incorporating non-traditional data sources.

 2. Strengths of the Manuscript

 The manuscript’s primary strength lies in its originality. Using expert podcasts as a qualitative dataset introduces a fresh perspective into financial market microstructure research. The methodological framework is clearly structured, and the sequential flow from qualitative extraction to lexicometric clustering and factor analysis is logically presented. The identification of five clusters—Institutional Infrastructure, Foundation and Transition, Market Efficiency and Resilience, Forecasting and Modelling, and Market Structure and Efficiency—demonstrates conceptual clarity. The theoretical grounding in classical microstructure models (inventory models and information asymmetry models) strengthens academic rigor.

 3. Methodological Evaluation

 While the methodology is innovative, several clarifications are required. The selection criteria for the 12 podcasts need stronger justification, particularly regarding representativeness and potential selection bias. The sample size may limit generalizability. The threshold of excluding keywords below 10% frequency should be theoretically justified. Further explanation is required on factor extraction decisions, including factor retention criteria and validation metrics. Inclusion of robustness checks (e.g., PCA comparison, cross-validation, or reliability statistics) would enhance rigor.

 4. Conceptual and Theoretical Contribution

 The study contributes conceptually by bridging practitioner discourse with academic microstructure theory. The clustering aligns well with established theoretical frameworks such as Glosten and Milgrom (1985), Ho and Stoll (1981), and information asymmetry literature. However, deeper integration between empirical findings and theoretical implications would improve coherence. Some interpretations appear descriptive rather than analytical. A stronger theoretical synthesis linking the three extracted factors with existing microstructure paradigms is recommended.

 5. Results and Interpretation

 The results are systematically presented, and figures such as the Zipf distribution, DHC dendrogram, and factor loading heatmap enhance clarity. However, interpretation of factor loadings requires more statistical explanation. The discussion sometimes overgeneralizes conclusions from qualitative discourse. A clearer distinction between exploratory insights and empirical validation would strengthen the findings.

 6. Limitations and Future Research

 The authors appropriately acknowledge limitations, including reliance on podcast transcripts and absence of empirical transaction-level validation. Future research directions are well-articulated. However, practical implementation pathways for policymakers and practitioners could be expanded further.

 7. Writing and Presentation

 The manuscript is generally well-organized. Minor grammatical inconsistencies and typographical issues should be corrected. Certain sections could be streamlined to avoid repetition, particularly in the discussion section.

 8. Final Recommendation

 The manuscript demonstrates originality and methodological innovation, but requires substantial clarification in methodological justification, theoretical integration, and statistical validation before it can be considered for acceptance. Addressing the methodological concerns and strengthening analytical depth would significantly enhance its scholarly contribution.

**Decision: Major Revision**

Is the work clearly and accurately presented and does it cite the current literature?

Partly

If applicable, is the statistical analysis and its interpretation appropriate?

Yes

Are all the source data underlying the results available to ensure full reproducibility?

Yes

Is the study design appropriate and is the work technically sound?

Partly

Are the conclusions drawn adequately supported by the results?

Partly

Are sufficient details of methods and analysis provided to allow replication by others?

Partly

Reviewer Expertise:

Accounting and Finance and block Chain technology

I confirm that I have read this submission and believe that I have an appropriate level of expertise to confirm that it is of an acceptable scientific standard, however I have significant reservations, as outlined above.

# Response

**Published:** 2026-05-23

**Authors:** Nitika Sharma

**Affiliations:** Chitkara University Chitkara Business School, Chandigarh, Chandigarh, India

Dear Reviewer,

 We sincerely thank you for your thorough and constructive evaluation of our manuscript. The insightful comments have helped us significantly strengthen the quality, rigor, and clarity of the work. We have carefully addressed each concern raised and have revised the manuscript accordingly. Below, we provide a point-by-point response to all comments. 

***Comment 1:** Podcast Selection Criteria and Sample Representativeness*

*Reviewer Comment: "The selection criteria for the 12 podcasts need justification particularly regarding representativeness and potential selection bias. The sample size may limit generalizability."*

**Response:** We really appreciate this observation. The cryptocurrency market microstructure podcasts selected for this study were chosen on purpose to capture authentic discussions about cryptocurrency. Our selection process was very structured. 

 We had four criteria for selecting the podcasts:

 * They had to be about market microstructure and cryptocurrency specifically.

 * The episodes had to feature people who work in the finance industry like traders or fund managers.

 * The podcasts had to be from known and authentic platforms.

 * They had to be popular and well-regarded by listeners.

 Regarding the idea that our sample size might be too small we know that this kind of research is meant to be exploratory and help us understand things better than being a statistically perfect representation of the whole population. The aim was to get an understanding of what the experts are saying, rather than make general statements about the whole population.

**Manuscript change: We added a section to the Methodology part of the manuscript that explains and justifies how we selected the podcasts and why we chose those specific ones.**

*Comment 2: Justification for the 10% Keyword Frequency Threshold*

*Reviewer Comment: "The threshold of excluding keywords below 10% frequency should be theoretically justified."*

**Response: **We thank the reviewer for pointing this out. We did not just choose the 10% threshold randomly. The pre-testing of thresholds, at 5%, 10%, 15% and 20% was done to see which one worked best. We looked at how the keywords clustered together and how easy they were to understand at each threshold. At 5% we got a lot of keywords that were not very important and made it hard to understand the clusters. At 15% and 20% we missed some keywords that helped us understand the expert discussions. The 10% threshold worked the best. It gave us a balance between having enough keywords and being able to understand them clearly.

**Manuscript change: We added a paragraph to the Data Analysis section that explains how we chose the 10% threshold and why it was the best choice.**

*Comment 3: Factor Extraction Decisions and Validation Metrics*

*Reviewer Comment: "Further explanation is required on factor extraction decisions, including factor retention criteria and validation metrics. Inclusion of robustness checks would enhance rigor."*

**Response:** A method called factor analysis was undertaken, which is a common way to identify patterns in data. We used clustered heatmap to decide how factors to keep. This criterion funnelled to three factors that have been included in the study. We also reported how much of the data each factor explained, which helps to understand how important each factor is.

**Manuscript change: We added additional and detailed information to the Results section regarding the analysis.**

*Comment 4: Depth. Linking Factors to Microstructure Paradigms*

*Reviewer Comment: "Deeper integration between findings and theoretical implications would improve coherence. Some interpretations appear descriptive than analytical."*

**Response: **We appreciate this comment. Agree that we needed to connect our findings to theoretical ideas more clearly. We mapped each of our three factors to established frameworks in the field of microstructure. The first factor is related to how institutions and regulations shape markets. The second factor is about how information affects market prices. The third factor is about how forecasting and volatility work in markets. We added a paragraph to the Discussion section that explains how these factors fit together into a bigger theoretical picture.

**Manuscript change: We revised the Discussion section to include theoretical explanations and connections to established ideas in the field.**

*Comment 5: Factor Loading Interpretation and Overgeneralization*

*Reviewer Comment: "Interpretation of factor loadings requires statistical explanation. The discussion sometimes overgeneralizes conclusions from discourse."*

**Response: **We thank the reviewer for this comment. We know that we needed to explain our factor loadings and avoid making general statements that were not supported by the data.

 We reported the loading values for each term on each factor and used a threshold to determine which loadings were meaningful. We also added a table that shows the terms for each factor and their loading scores. We revised the discussion to qualify our claims and avoid overgeneralizing. We made sure to say that our conclusions were based on expert discourse.

**Manuscript change: We added a table to the Results section that shows the factor loadings and revised the Discussion section to be more careful in the interpretation, implication and discussion.**

*Comment 6: Writing Quality and Practical Implications*

*Reviewer Comment: "Minor grammatical inconsistencies and typographical issues should be corrected. Certain sections could be streamlined to avoid repetition. Practical implementation pathways for policymakers and practitioners could be expanded further."*

**Response: **We went through the manuscript and corrected any grammatical or typographical errors.

 We also revised the Discussion section to make it more relevant and detailed.

 We added a section that outlines some practical implications of our findings for policymakers and practitioners. 

**Manuscript change: We proofread the manuscript and made corrections. We also added a section to the Discussion section that outlines practical implications.**

 We hope that the revisions adequately address the reviewer's concerns and that the manuscript is now suitable for acceptance. We remain grateful for the opportunity to improve this work and are happy to provide any further clarification.
